# Neutrophil-to-lymphocyte ratio and neurological deterioration following acute cerebral hemorrhage

**DOI:** 10.18632/oncotarget.15423

**Published:** 2017-02-16

**Authors:** Simona Lattanzi, Claudia Cagnetti, Leandro Provinciali, Mauro Silvestrini

**Affiliations:** ^1^ Department of Experimental and Clinical Medicine, Neurological Clinic, Marche Polytechnic University, Ancona, Italy

**Keywords:** cerebrovascular disease, intra-cerebral hemorrhage, lymphocyte, neutrophil, neutrophil-to-lymphocyte ratio

## Abstract

Immunity plays key roles in pathophysiology of intracerebral hemorrhage (ICH). The aim of the study was to determine whether the peripheral leukocyte count and neutrophil-to-lymphocyte ratio (NLR) predicted neurological deterioration (ND) after ICH. We identified consecutive patients with ICH who had blood sampling performed within 24 hours from symptom's onset. Total white blood cells (WBC), absolute neutrophil count (ANC) and absolute lymphocyte count (ALC) were retrieved, and the NLR computed as the ratio of the ANC to ALC values. The study endpoint was the occurrence of neurological deterioration (ND) within 7 days after ICH. One hundred ninety-two subjects were enrolled, whose 54 (28.1%) presented ND. At multivariate analysis, the WBC (adjusted odd ratio [_adj_OR] for 1,000 leukocytes increase 1.29, 95% confidence interval [CI] 1.11-1.50), ANC (_adj_OR for 1,000 neutrophils increase 1.61, 95% CI 1.30-1.99), ALC (_adj_OR for 1,000 lymphocytes increase 0.21, 95% CI 0.09-0.49) and NLR (_adj_OR for 1-point increase 1.65, 95% CI 1.36-2.00) were independently associated with ND (p≤0.001). The NLR resulted the best discriminating variable for the occurrence of the adverse outcome (area under the curve 0.888, 95% CI 0.832-0.945; *p* < 0.001). The NLR predicted ND after acute ICH and can aid in the risk stratification of patients.

## INTRODUCTION

Spontaneous intracerebral haemorrhage (ICH) represents approximately 10% to 15% of all strokes and affects over 1 million people per year worldwide. It is characterized by high rates of mortality and residual disability among survivors, and currently no therapeutic strategies have demonstrated definitive benefit [1]. Neurological deterioration (ND) is common after ICH and it is associated with increased length of in-hospital stay, poor functional recovery and death [2]; notwithstanding, reliable and easy-to-use predictors allowing the early identification of unstable at-risk patients are not well established.

Immune reaction is a major feature of ICH pathology and influences its course; the response to cerebral hematoma is not bounded to the brain and results in systemic effects, and inflammatory markers on admission, such as fever, elevated leukocyte count, interleukin-6 and C-reactive protein are associated with worse prognosis [3–5]. The neutrophil-to-lymphocyte ratio (NLR), an easily available synthesis of the inflammatory levels and enhanced immune pathways, is associated to 3-month recovery in ICH patients [6, 7], but its link with short term outcome is unknown. The aim of this study was to evaluate the relationship between the total and differential leukocyte counts and the NLR at admission with the occurrence of ND during the initial week after ICH onset.

## RESULTS

**Figure 1:**
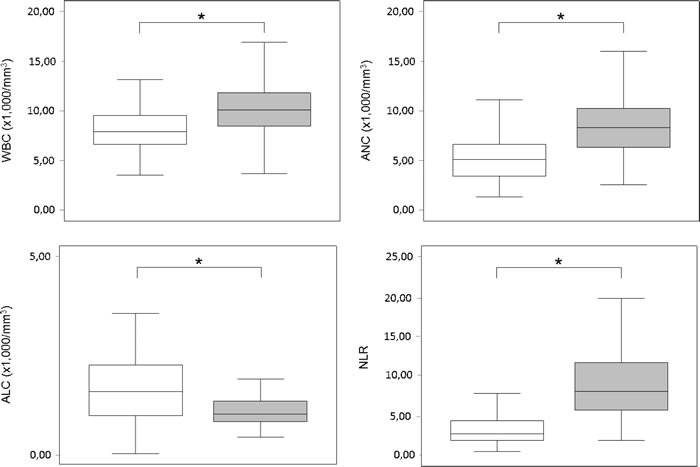
Leukocyte counts and neutrophil-to-lymphocyte ratio Box plots showing leukocytes counts and NLR according to neurological deterioration within the initial week after ICH (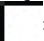 = ND, 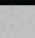
= Non-ND; * = *p* < 0.001). Abbreviations: ALC = absolute lymphocyte count; ANC = absolute neutrophil count; CI = confidence interval; ICH = intra-cerebral haemorrhage; ND = neurological deterioration; NLR = neutrophil-to-lymphocyte ratio; WBC = white blood cells.

**Figure 2:**
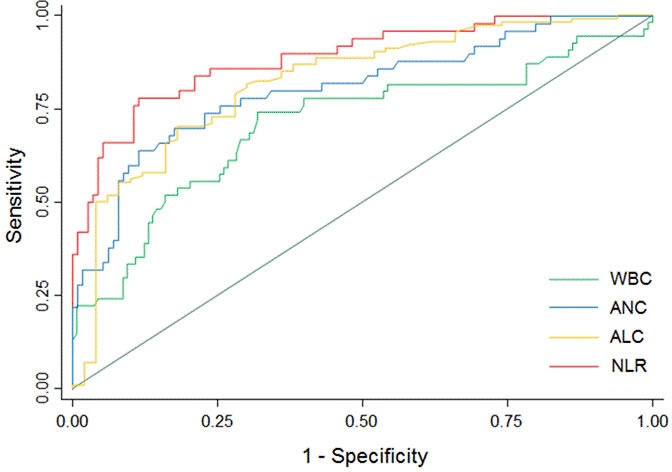
Receiver operating characteristic curves for neurological deterioration prediction Predictive values of WBC, ANC and NLR for ND and of ALC for non-ND. Area under the curve 0.709 (95% CI, 0.618-0.799; SE = 0.046) for WBC; 0.807 (95% CI, 0.730-0.884; SE = 0.039) for ANC; 0.821 (95% CI, 0.751-0.893; SE = 0.036) for ALC; 0.888 (95% CI, 0.832-0.945; SE = 0.029) for NLR (*p* values <0.001). Abbreviations: ALC = absolute lymphocyte count; ANC = absolute neutrophil count; CI = confidence interval; ND = neurological deterioration; NLR = neutrophil-to-lymphocyte ratio; SE = standard error; WBC = white blood cells.

A total of 192 patients were recruited, whose 54 (28.1%) presented ND during the first week after ICH onset; univariate comparisons of baseline characteristics between the outcome patient groups are summarized in Table 1. The patients who worsened had higher WBC (10.80 ±4.96 versus 8.04±2.22; *p* < 0.001), higher ANC (9.14 ±4.77 versus 5.31±1.94; *p* < 0.001), lower ALC (1.15 ±0.63 versus 2.04±1.86; *p* = 0.001), and higher NLR (9.46±5.80 versus 3.28±1.98; *p* < 0.001) compared to those who did not experience ND (Figure 1). The WBC was strongly correlated to the ANC (Spearman rho 0.920; *p* < 0.001); the WBC, ANC and ALC were weakly to moderately correlated to the baseline ICH volume (Spearman rho 0.254, 0.356 and -0.277, respectively; *p* ≤ 0.001) and initial NIHSS score (Spearman rho 0.228 [*p* = 0.003], 0.344 [*p* < 0.001] and -0.315 [*p* < 0.001], respectively). After logistic regression analysis, the WBC, ANC, ALC and NLR were all significantly associated (p≤0.001) with ND (Table 2). To remove skew and minimize the influence of extreme values, baseline ICH volume was log transformed after addition of the value 1.1 mL to eliminate negative values. None of the multivariate models suffered from collinearity (variance inflation factors ranged from 1.02 to 2.21). The model performance for ND prediction improved when laboratory parameters were added as continuous variables (without: LR (8) 55.068, BIC’ -13.008; with WBC: LR (9) 70.548, BIC’ -23.231; with ANC: LR (9) 80.435, BIC’ -34.536; with ALC: LR (9) 69.409, BIC’ -23.510; with NLR: LR (9) 98.072, BIC’ -52.173); the NLR had a better additional predictive value compared to WBC, ANC and ALC. At the ROC analysis with respect to ND, the area under the curves were 0.709 (95% CI, 0.618-0.799; SE = 0.046), 0.807 (95% CI, 0.730-0.884; SE = 0.039), 0.821 (95% CI, 0.751-0.893; SE = 0.036) and 0.888 (95% CI, 0.832-0.945; SE = 0.029) for the WBC, ANC, ALC and NLR, respectively (Figure 2). The Youden's index identified the best cut-off of NLR for ND at 5.46 [sensitivity 70.4% (95% CI, 58.2-82.5%), specificity 90.6% (95% CI, 85.7-95.5%), positive predictive value 74.5% (95% CI, 62.5-86.5%), negative predictive value 88.7% (95% CI, 83.4-93.9%), positive likelihood ratio [LR] 7.47 (95% CI, 4.33-12.89), negative LR 0.33 (95% CI, 0.22-0.50), accuracy 84.9% (95% CI, 79.8-90.0%)].

**Table 1:** Baseline characteristics according to neurological deterioration

	Full cohort (*n*= 192)	ND (*n* = 54)	Non-ND (*n*= 138)	*P* value
**Demographics** Age (years) Female sex Caucasian	66.9 (12.5) 69 (35.9) 187 (97.4)	68.0 (12.6) 17 (31.5) 53 (98.1)	66.4 (12.4) 52 (37.7) 134 (97.1)	0.429^a^ 0.421^b^ 0.682^b^
**Clinical history** Hypertension Diabetes mellitus Hyperlipidemia Atrial fibrillation Coronary artery disease Prior stroke/TIA Current smoker	123 (64.1) 40 (20.8) 65 (33.9) 19 (9.9) 23 (12.0) 15 (7.8) 39 (20.3)	31 (57.4) 11 (20.4) 18 (33.3) 7 (13.0) 7 (13.0) 5 (9.3) 15 (27.8)	92 (66.7) 29 (21.0) 47 (34.1) 12 (8.7) 16 (11.6) 10 (7.2) 24 (17.4)	0.229^b^ 0.921^b^ 0.924^b^ 0.373^b^ 0.793^b^ 0.640^b^ 0.108^b^
**Pre stroke medications** Antihypertensive drugs Statins Antiplatelet agents Oral anticoagulants	101 (52.6) 58 (30.2) 40 (20.8) 18 (9.4)	27 (50.0) 15 (27.8) 15 (27.8) 7 (13.0)	74 (53.6) 43 (31.2) 25 (18.1) 11 (8.0)	0.651^b^ 0.646^b^ 0.138^b^ 0.286^b^
**Clinical assessment** Systolic BP (mmHg) Diastolic BP (mmHg) NIHSS score Systolic BP variability (CV) Diastolic BP variability (CV)	150 (135-170) 80 (75-90) 9 (6-14) 10.1 (4.7) 10.9 (4.0)	155 (135-175) 80 (75-90) 13 (9-18) 11.6 (4.4) 12.0 (4.1)	150 (135-165) 80 (75-90) 8 (5-12) 9.5 (4.6) 10.6 (3.9)	0.422^c^ 0.998^c^ <0.001^c^ 0.004^b^ 0.026^b^
**Brain imaging of ICH** Volume (mL) Location *Lobar* *Deep* *Brainstem* *Cerebellum* Intraventricular extension	8.1 (3.5-16.0) 63 (32.8) 119 (62.0) 1 (0.5) 9 (4.7) 44 (22.9)	16.4 (8.1-27.7) 14 (25.9) 39 (72.2) 0 (0.0) 1 (1.9) 19 (35.2)	5.9 (2.6-13.0) 49 (35.5) 80 (58.0) 1 (0.7) 8 (5.8) 25 (18.1)	<0.001^c^ 0.204^b^ 0.067^b^ 0.531^b^ 0.245^b^ 0.011^b^
**Time onset-to-sample (hours)**	17.3 (15.7-19.3)	17.0 (15.7-18.4)	17.4 (15.7-19.8)	0.449^c^

Data are mean (SD) or median (IQR) for continuous variables, and n (%) for categorical variables.

^a^Two-sample t test. ^b^Chi-squared test. ^c^Mann-Whitney test.

Abbreviations: BP = blood pressure; CV = coefficient of variation; ND = neurological deterioration; NIHSS = National Institute of Health Stroke Scale; TIA = transient ischemic attack.

**Table 2:** Associations of leukocyte counts and neutrophil-to-lymphocyte ratio with neurological deterioration

Independent Variable	Unadjusted		Adjusted*	
	OR (95% CI)	*p* value	OR (95% CI)	*p* value
White blood cells	1.33 (1.17-1.51)	<0.001	1.29 (1.11-1.50)	0.001
Absolute neutrophil count	1.66 (1.38-2.00)	<0.001	1.61 (1.30-1.99)	<0.001
Absolute lymphocyte count	0.14 (0.07-0.30)	<0.001	0.21 (0.09-0.49)	<0.001
Neutrophil-to-lymphocyte ratio	1.76 (1.47-2.11)	<0.001	1.65 (1.36-2.00)	<0.001

ORs for every 1,000 white blood cells, neutrophils or lymphocytes and 1-point NLR increases are obtained with logistic regression analysis.

*Adjustment by age, sex, initial NIHSS score, baseline ICH volume (log transformed), hematoma location, presence of intraventricular hemorrhage, systolic and diastolic BP variability.

Abbreviations: BP=blood pressure; CI = confidence interval; NIHSS = National Institute of Health Stroke Scale; OD = odds ratio.

## DISCUSSION

The main results of our study are that higher neutrophils and lower lymphocytes upon admission independently predicted ND during the initial week after ICH onset, and the NLR represented a reliable predictive biomarker.

Inflammation takes place soon after the ICH and in its early stage is mainly sustained by polimorphonuclear activation. Neutrophils represent the first leukocytes to actively migrate since the first hours after ICH from peripheral blood into the brain, and promote secondary injury. The infiltrating leukocytes release inflammatory and cytotoxic mediators which enhance the peri-lesional edema by favoring capillary permeability, cell swelling and blood-brain barrier damage [8, 9]. The capsule-like granulation tissue that develops around the bleeding increases the hematoma tension and favors the intra-lesional edema [10]. The damage to endothelium and basal lamina induced by the inflammatory cascade and blood leaks from the friable capillaries of the granulation tissue contribute to hematoma enlargement [10]. Hence, edema and hematoma growth favored by enhanced neuro-inflammatory pathways may reasonably increase the intracranial pressure, favor the mass effect and cerebral tissue displacement, and drive to ND. Accordingly, the neutrophil depletion has been shown to attenuate blood brain barrier breakdown and vessel permeability, decrease macrophage responses and astrocytes’ activation, and finally reduce secondary brain damage in animal stroke models [11].

Lymphocytes are major regulators of immunity and their reduction and functional deactivation are signatures of brain damage which occur as early as 12 hours after stroke as an effect of hyperactivity of sympathetic nervous system and hypothalamic-pituitary adrenal axis [12]. Nonetheless, the role of lymphocytes in ND after ICH has been underappreciated. As key players of cellular and humoral responses, lymphocytes are crucial for the host defense against pathogens. In experimental studies, post-stroke inhibition of adaptive immunity resulted in spontaneous bacterial infections, and low percentages of lymphocytes have been independently linked to increased incidence of infections in ICH patients [13, 14]. In-hospital infections are among the most common medical complications encountered after stroke and can worsen the clinical course by favoring hypoxia, acidosis, electrolytic unbalance and venous thromboembolism, inducing hyperthermia and increasing cerebral metabolic demands [15]. Additionally, specific subsets of regulatory lymphocytes modulate immunity and exert protective effects by limiting the autoreactive cells and the inflammatory-induced damage at the cerebral sites [16].

The NLR was independently associated to the occurrence of ND and outperformed the leukocytes counts as predictors of clinical worsening. The NLR is a composite inflammatory index that integrates information on the innate and adaptive pathways. In acute ICH, it could represent a surrogate systemic bio-marker of degree and direction of the immune response to hematoma. Despite obvious limits for any time-based classification, the drivers of ND are heterogeneous and both intra-cerebral and systemic conditions play different roles according to the worsening time-point [17]. Blood amount and hematoma enlargement are mainly associated to deterioration within 24 hours after ICH, edema formation and expansion are more likely to contribute between days 1 to 3 from onset, while infections and medical complications are the main leading entities thereafter and up to two weeks [18]. Accordingly, the NLR could synthetize at once the susceptibility to the secondary brain damage and post-stroke complications which, favored by the increase of neutrophils and reduction of lymphocyte, could promote ND after ICH.

The study should be interpreted in the light of some limitations. The retrospective design and the performance of all testing as for real-word practice and physician's judgment allowed to describe associations and raise working hypotheses. The relationships between immune response and ND can be definitively understood only through prospective studies based upon pre-specified time points for blood sampling and imaging. Further, findings could not be extended to hyper-acute clinical worsening according to study protocol and inclusion of patients up to 24 hours post stroke. The main study strengths allowed generalizability of results to everyday clinical setting and included the enrollment of patients regardless of ICH location, the wide availability of all laboratory variables, and the cost-effectiveness of the NLR as a readily accessible biomarker.

In conclusion, our findings suggested a simple, inexpensive and easily available tool to early identify patients at increased risk of clinical deterioration. Neurological stability across the initial week after stroke onset translates into low risks of long-term morbidity and mortality [17], and the prediction of ND may be of aid to design clinical management and assist patient prognostication. The understanding of the underlying inflammatory pathways warrants further investigations and may help to discover new targets for neuroprotection and novel strategies to improve clinical outcome.

## MATERIALS AND METHODS

### Participants and study outcome

We retrospectively identified consecutive patients hospitalized at the Stroke Unit of the Marche Polytechnic University, Ancona, Italy from January 2008 to July 2016 for stroke syndrome due to acute spontaneous ICH who underwent admission routine blood sampling and cranial CT neuroimaging within 24 hours from symptom onset. Demographics, medical history, Glasgow Coma Scale (GCS) and National Institutes of Health Stroke Scale (NIHSS) [19] scores at admission and clinical examinations, admission blood pressure (BP) and 24-hours BP variability by means the coefficient of variation [20–23] were retrieved. Total white blood cells (WBC), absolute neutrophil count (ANC) and absolute lymphocyte count (ALC) were collected from admission blood work; the NLR was computed as the ratio of the ANC to ALC values. Baseline volume, topography (lobar, deep, brainstem, cerebellum) and intraventricular extension (presence versus absence) of ICHs were determined. All CT scans were read by a single evaluator blinded to clinical and biochemical data. The previously validated ABC/2 or ABC/3 methods were used to estimate the hematoma volume for round and ellipsoid or irregularly and separately shaped haemorrhages, respectively [24]. These methods correlated well with more sophisticated planimetric volume measurements [25]. The ICH was considered as lobar when it predominantly involved the cortical or sub-cortical white matter of cerebral lobes or as deep when it was limited to the internal capsule, basal ganglia or thalamus [26]. All patients received standard management according to current national guidelines for stroke [27]; CT angiography or conventional cerebral angiography were performed to assess the presence of any structural parenchymal or vascular abnormality.

The outcome measure was the occurrence of neurological deterioration (ND) defined as a 4 point or greater increase in the NIHSS score or 2 point or greater decrease in the GCS or death from the time of admission to 7 days post-hemorrhage [18].

Patients presenting with isolated intraventricular hemorrhage, hemorrhage secondary to brain tumor, dural venous sinus thrombosis, ruptured arteriovenous malformation or aneurysm and patients receiving immunomodulatory treatment (e.g. corticosteroids, azathioprine, methotrexate, other cytostatic and biologicals agents as monoclonal antibodies) before admission were not considered for the study.

### Statistical analysis

Values are presented as mean ± SD or median (interquartile range [IQR]) for continuous variables and as the number (percent) of subjects for categorical variables. Comparisons were made through the Student t test, Mann-Whitney test or Chi-squared test as appropriate. Spearman correlation was used to correlate continuous variables. The associations between the WBC, ANC, ALC, NLR and the study endpoint were determined using logistic regression models; the variables with *p* values < 0.05 from comparison of baseline characteristics and selected variables (age, sex, initial NIHSS score, baseline volume, location and intraventricular extension of ICH) [28] were forced into multivariate analysis. Overall fit of the models with and without the laboratory parameters (WBC, ANC, ALC, NLR) was assessed by the likelihood ratio (LR) χ^2^ (1-degree-of freedom) test and Bayesian Information Criterion (BIC’). Higher values of LR and lower values of BIC’ imply better fit. The receiver operating characteristic (ROC) analysis was performed to evaluate the ability of the WBC, ANC, ALC and NLR to predict ND. Separate models were constructed for the WBC, ANC, ALC and NLR. The collinearity between exposure variables was assessed with the variance inflation index. Results were considered significant for *p* values < 0.05 (two sided). Data analysis was performed using STATA/IC 13.1 statistical package (StataCorp LP, Texas, USA).
